# 

**Published:** 1828-01-01

**Authors:** 


### CONTENTS

OF THE

### MEDICO-CHIRURGICAL REVIEW.

### No. XV. JANUARY 1, 1828.

(*First Department.*)

ANALYTICAL REVIEWS.

ART. I.

Malaria; an Essay on the Production and Propagation of this Poison, and on the Nature and Localities of the Places by which it is produced, at Home and Abroad. By JOHN MACCULLOCH, M.D. F.R.S. &c. &c. .......... 1

II.

Rambling Notes and Reflections, suggested during a Visit to Paris in the Winter of 1826-7. By Sir ARTHUR BROOKE FAULKNER, M.D. &c. .... 27

1. Satyrical Portrait of the English Medical Profession generally.... 28
2. Illiberal Caricature of the "GENERAL PRACTITIONER" ........ 30
3. Sir Arthur's Vindication of the College of Physicians .......... 32
4. Strictures on this Vindication .......... 32
5. Illegality and Absurdity of the Oath administered by the College 33
6. Declaration of Sir Arthur, that the FELLOWS of the College consult with SURGEONS and APOTHECARIES only as they would with NURSES! .......... 33
7. Sir Arthur's Reasons for not prosecuting Quacks .......... 34
8. His Plan of Medical Reform, and Eulogy on the College......... 36

III.

Clinical Observations on the Efficacy of Hydro-Chloruret of Lime, as a Remedy in certain Stages of Fever and Dysentery. By ROBERT REID, M.D. 38

IV.

Traité sur les Gastralgies et les Enteralgies; ou Maladies de l'Estomac et des Intestins. Par M. BARRAS, M.D. &c. .......... 45

V.

Observations upon the Origin and Latent Period of Fever. By HENRY MARSH, M.D.—(Dublin Hospital Reports, Vol. IV.) .......... 55

VI.

M. ANDRAL, Jun. on Peritoneal Inflammation, Chronic and Acute. From the Hospital Practice of LA CHARITE .......... 66

VII.

Memoirs of West Indian Fevers. By JAMES WILSON, M.D. .......... 74

VIII.

Dr. BRIGHT's Reports of Medical Cases in Guy's Hospital .......... 90

REPORT 1. On Dropsical Effusions, as connected with Disease in the Kidneys .......... 92

IX.

Dr. FORBES's new Translation of the second Edition of Laennec on Auscultation 103

X.

Medical Botany .......... 117

### Quarterly Periscope.

### PART I.

### HOSPITAL REPORTS.

1. M. Bayle on the Influence of the Stomach on the Mind—and of the Influence of the Mind in disordering the Stomach.—*(Charenton.)* .......... 121

2. On Diseases of the Female Mamma, collected at St. George's, and other Metropolitan Hospitals.......................................... 126

3. Cases of Internal Strangulation.—*(La Charité.)*...................... 129

4. Dr. Duncan on Empyema and Pneumo-Thorax.—*(Royal Infirmary.)* .... 131

A. State of Auscultation in England and Scotland ..............

B. Remarkable Instance of erroneous Judgment, lately given by M. Portal, in the Case of an English Gentleman................ 132

C. Various Cases of Empyema and Pneumo-Thorax.............. 133

5. Dissection of an Epileptic Patient.—*(Bicêtre.)* ..................... 136

6. Case of Empyema, with Baron Larrey's Operation.—*(Garde Royale.)*.... 137

7. Dr. Chambers on Pulmonary Abscess.—*(St. George's Hospital.)* ........ 138

8. M. Broussais' Hospital Report.—*(Val de Grace.)* .................... 142

Comparison of the Success of the New and Old Doctrines.............. 142

1. On Pleuritis ................................................ 142

2. On Acute Bronchitis ......................................... 143

3. On Pneumonia, Acute.......................................... 144

4. Chronic Bronchitis .......................................... 145

5. Acute Gastro-Enteritis ...................................... 145

6. Acute Duodenitis............................................. 147

7. Colitis ...................................................... 147

9. Curious Species of Cerebral Hemorrhage.—*(Bicêtre.)* ................ 148

10. Fatal Sickness from Pregnancy.—*(Hotel Dieu.)*...................... 149

11. Peculiar Disease (False Aneurism) of the Heart.—*(La Charité.)* .. ..... 151

12. M. Buet on Intestinal Invaginations.—*(Hotel Dieu.)*................. 154

13. On Compression in Articular Inflammation.—*(Brussels Hospital.)* ...... 156

14. M. Martin on Endermic Medication.—(*Hotel Dieu.*) ..................... 158

15. An Essay on Gymnastic Exercises.—(*Original and Compiled.*).......... 159

16. Successful Ligature of the Subclavian Artery, by Robert Thorpe, Esq.—(*Manchester Infirmary.*) ...................................... 164

16. Curious Case of Morbid Sensibility of Half the Body.................. 166

18. Interesting Case of Angina Suffocans.—(*Hotel Dieu.*) ................ 168

19. Stricture of the Œsophagus—Curious Error in the Report.—(*Hospital of Surgery.*) ...................................................... 169

20. Remarkable Case of Hernia, with the Dissection.—(*Bartholomew's.*) .... 170

21. Aneurismal Pouch (False Aneurism) of the Heart.—(*St. Thomas's Hospital.*) 174

22. Fatal Œdema of the Glottis.—(*Guy's Hospital.*)......................... 175

23. Fracture and Depression of the First Dorsal Vertebra.—(*Guy's Hospital.*) 176

24. Memoir on Organic Diseases of the Encephalon. By M. Rackem, M.D.—(*Hôpital de Voltera.*).......................................... 178

25. Remarkable Disease of the Epiploon ................................ 184

26. Chloruret of Lime in Purulent Ophthalmia ........................... 184

### Quarterly Periscope.

### PART II.

### GENERAL REVIEW OF PERIODICALS.

1. Rheumatism—Spinitis, with the Dissection .......................... 185
Illiberal Conduct of one Medical Man to another ..................... 185
2. Case of Hypochondriasis cured by an Ague ............................ 186

3. Case of Gangrene of the Feet from obstructed Aorta ........ 187
4. Pericarditis, and Hypertrophy of the Heart in a Child ........ 188
5. Epidemic of Groningen........ 189
6. Gastro-Cerebral Inflammation, with Symptoms of Hydrophobia........ 191
7. Mr. Iliff's Case of "Spontaneous Paraplegia," with Remarks ........ 192
8. Professor Schonlein on Pneumo-Phthisis Cyanotica........ 193
9. Fatal Case of Lithotomy ........ 194
10. Essay on Extirpation of the Uterus, general and partial, by Professor Gustavus Hesse, with many Cases........ 195
11. Diseases of the Nail ........ 200
12. Case of Fracture of the Spine, at Guy's Hospital, (with Remarks) ...... 201
13. M. Paillard on Enlargement of the Upper Lip........ 202
14. Baron Larrey's new Method of treating Fractures ........ 202
15. M. Lambert on introducing Medicines by the Skin ........ 203
16. General Paralysis from Contusion in the Spine.—(M. Broussais) ....... 203
17. Case of remarkable Nævus Maternus, by Mr. Bennett ........ 204
18. Dr. Hare's Formula for Denarcotised Laudanum ........ 205
19. Professor Broussais's Opinion of English Medicine........ 205
——— Criticisms on the Edinb. Journ. Med. Science...... 205
——— on Dr. Brown, of Sunderland........ 205
——— on Mr. Travers........ 205
——— on Dr. Gairdner ........ 205
——— on Mr. Bell ........ 206
20. Curious Instance of Blindness from a Blow on a Branch of the Fifth Pair 206
21. Professor Dzondi's new Method of treating Syphilis........ 206
22. Mr. Kingsley on Purpura Hæmorrhagica ........ 207
23. Case of Bronchotomy for the Liberation of a Grain of Corn ........ 208
24. Professor Mayer's Experiments on Ablation of the Kidneys........ 208
25. Exposé of the various Experiments on Digestion, by Messrs. Tiedemann, Gmelin, Leuret, and Lassaigne ........ 209
26. Pythagoras Redivivus ........ 213
27. The Instrument of Justice; or, Liberality of the Lancet, in the Case of Mr. Mayo, of the Winchester Hospital ........ 214
28. New Experiments on Section of the Pneumo-gastric Nerves, made at the Veterinary School of Alfort.—By M. Dupuy........ 215
Pemphegoid Affection of the Spleen, containing Matter capable of producing (by Inoculation) the same Disease of Spleen in other Animals.. 216
29. Fatal Exhalation of Blood into the Stomach ........ 216
30. Remarkable Case of Catalepsy........ 217
31. On the Treatment of Erysipelas by Incisions ........ 218
32. New Remedy (Insufflation of Air) for Asthma ........ 220
33. Ingestion of Hot Water for the Cure of Gout ........ 221
34. Inflammation of the Neck of the Bladder, with Strictures on the Bougie.. 222
35. Case of Luxation of the Cervical Vertebræ........ 222
36. Remarkable Case of Emphyema of the Substance of the Heart, &c. .... 223
37. Operation for Emphysema, twice in the same Person, after a long Interval, 224
38. Perforation of the Intestinum Ileum ........ 225
39. Gangrene of the Lungs........ 226
40. Signs of the Times, or Epidemics of the Day—Venesection—Dissection—Inspection ........ 227
41. Phlebitis........ 231
42. Extra-Uterine Pregnancy........ 231
43. Duration of Pregnancy........ 232

### MEDICINA FORENSICA.

I. The Royal College of Physicians.
Petition or Remonstrance presented to the College 33 years ago, signed by 14 eminent Physicians—some of them still living, and embodying most of the Grievances now complained of........ 233

II. Discussion on Medical Education ........................... 237
Arguments for and against the System of Apprenticeship............ 237
III. On the Dissensions in the University of Edinburgh.................. 239
Will Greek and Algebra cure Diseases? ........................... 241
IV. Late Regulations of the Apothecaries' Company, with Remarks ...... 242
V. Lunacy—Insanity—Unsound Mind. Dr. Haslam examined.......... 243
VI. Observations on Lunatic Asyla .................................. 246
VII. Medico-Chirurgical Society—Mendacity of the Lancet............. 247
VIII. College of Physicians *versus* Dr. Harrison ........................ 248

### PRIZE-HOSPITAL REPORT, No. V.

*Mr. Wickham, Guy's Hospital.*

1. Delirium Tremens ending in Idiotism ................................ 249
2. Epilepsy from Onanism ............................................ 250
3. Dr. Elliotson's Treatment of Fever ................................ 250
4. Sloughing Venereal Sores.......................................... 251
5. Extirpation of a Fungoid Tumour .................................. 253
6. Fungoid Disease of the Hip ....................................... 254
7. Dislocation of the Radius forwards ................................ 255
8. Protrusion of the Fundus of the Bladder........................... 256
9. Fatal Case of Cut Throat .......................................... 257
10. Spasmodic Contraction of the Thumb.............................. 257
11. Various Cases of Hernia .......................................... 258
12. Fatal Case of Bronchitis ......................................... 260
13. Lithotomy ........................................................ 261
14. Wound of the Brachial Vein, &c. .................................. 262
15. Icthiosis—Wound of Brachial Vein................................. 263
16. Diffuse Inflammation of Cellular Tissue .......................... 263
17. Compound Fracture of the Tibia and Fibula ....................... 264
18. Case of Poisoning ................................................ 265
19. Fungus of the Brain .............................................. 266

### EXTRA-LIMITES.

Dr. Mackintosh's Strictures on Cullen's Doctrines, as taught in the Edinburgh University.......................................................... 273

Postsctipt to No. XV.

Mr. Wardrop's Case of Mrs. Denmark, on whom the Operation of tying the Subclavian for internal Aneurism was performed, examined, with Remarks on Mr. W's Statements ................................................ 281

### INTELLIGENCE, &c.

Dr. Cooke ........................................................... 283
Mr. Guthrie's Action against the Lancet ............................. 283
Literary Notices .................................................... 283

Address to the Subscribers of the Medico-Chirurgical Review.............. 284

Prize-List, &c. ...................................................... 288

